# Acoustic tweezing cytometry for mechanical phenotyping of macrophages and mechanopharmaceutical cytotripsy

**DOI:** 10.1038/s41598-019-42180-3

**Published:** 2019-04-05

**Authors:** Xiaowei Hong, Phillip M. Rzeczycki, Rahul K. Keswani, Mikhail D. Murashov, Zhenzhen Fan, Cheri X. Deng, Gus R. Rosania

**Affiliations:** 1 Department of Biomedical Engineering, University of Michigan, Ann Arbor, MI 48109 USA; 2 Department of Pharmaceutical Sciences, University of Michigan, Ann Arbor, MI 48109 USA; 3 Department of Mechanical Engineering, University of Michigan, Ann Arbor, MI 48109 USA

## Abstract

Macrophages are immune cells responsible for tissue debridement and fighting infection. Clofazimine, an FDA-approved antibiotic, accumulates and precipitates as rod-shaped, crystal-like drug inclusions within macrophage lysosomes. Drug treatment as well as pathophysiological states could induce changes in macrophage mechanical property which in turn impact their phenotype and function. Here we report the use of acoustic tweezing cytometry as a new approach for *in situ* mechanical phenotyping of macrophages and for targeted macrophage cytotripsy. Acoustic tweezing cytometry applies ultrasound pulses to exert controlled forces to individual cells via integrin-bound microbubbles, enabling a creep test for measuring cellular mechanical property or inducing irreversible changes to the cells. Our results revealed that macrophages with crystal-like drug inclusions became significantly softer with higher cell compliance, and behaved more elastic with faster creep and recovery time constants. On the contrary, phagocytosis of solid polyethylene microbeads or treatment with soluble clofazimine rendered macrophages stiffer. Most notably, application of ultrasound pulses of longer duration and higher amplitude in ATC actuated the integrin-bound microbubbles to mobilize the crystal-like drug inclusions inside macrophages, turning the rod-shaped drug inclusions into intracellular microblender that effectively destructed the cells. This phenomenon of acoustic mechanopharmaceutical cytotripsy may be exploited for ultrasound activated, macrophage-directed drug release and delivery.

## Introduction

Macrophages are phagocytic cells of the immune system that reside in all adult tissues. They play a central role in many biological processes, including development, metabolic regulation, and defense against invading pathogens^1^. Macrophages are highly motile and move within tissues. They are capable of ingesting various particles for tissue debridement during wound healing and regeneration as well as for defending organisms against invading pathogens^2^. Biomechanical models of cell structure and function suggest that viscoelastic property of macrophages affects their migration and ability for particle engulfment. Changes in mechanical properties of macrophages in response to stimuli in their chemical and physical microenvironment not only underlie pathophysiological states but also impact macrophage phenotype and function^3^. Alterations in cytoplasmic viscoelasticity of macrophages may result from changes in cytoskeletal organization due to intracellular accumulation of exogenous particles in macrophage lysosomes. In turn, changes in cytoplasmic viscoelasticity may impact cellular deformability, phagocytic ability, and cell motility^4^. It has been recognized that macrophage elasticity is a major determinant of innate macrophage function^5^.

Clofazimine (CFZ) is an FDA-approved, small molecule drug and has been used to treat leprosy for decades as an effective antibiotic^6^. It has also been investigated as a potential candidate to overcome resistant tuberculosis (TB), particularly if they can be targeted to the infection site^7^. Long term treatment results in CFZ accumulation and precipitation inside macrophage lysosomes where it self-assembles to form elastic, strut-like structures, referred as crystal-like drug inclusions (CLDIs)^8,9^. While soluble CFZ is cytotoxic, formation of CLDIs renders macrophages as massive drug depots, sequestering the drug^10^. CFZ CLDIs have been characterized as ordered supramolecular structures that are stabilized by the pH and chloride concentrations present in macrophage lysosomes^11^, yet it is unclear how these CDLIs alter mechanical properties of macrophages and affect macrophage function, given the relative large size and structure of these CLDIs. In addition, little is known about how efficacy and safety profile of the drug are affected by its precipitation, self-assembly, and intracellular accumulation within macrophages.

Therefore in this study, we aim to mechanically characterize macrophages with CLDIs. Macrophage-stabilized CFZ biocrystals were isolated from CFZ treated mice. The isolated CFZ crystals were then incubated with naïve macrophages *in vitro* to be readily ingested, resulting in direct loading of the CFZ crystals into the macrophages^12^. The CFZ biocrystals are biocompatible and stably retained within the live macrophagesv, providing a unique opportunity for examining the impact of CLDIs on mechanical property of macrophages.

**Figure 1:**
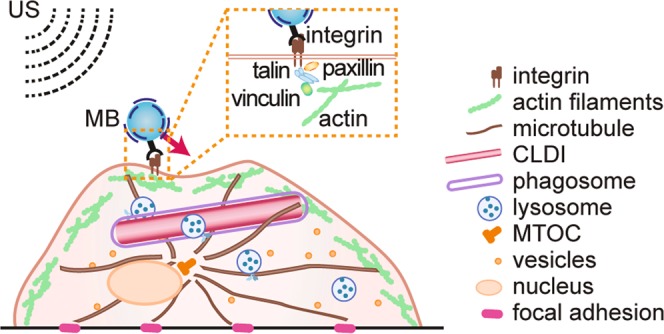
RGD-coated lipid microbubbles (MBs) attached to macrophages via RGD-integrin binding in acoustic tweezing cytometry (ATC). A phagocytosed CLDI is depicted to nest within the architecture of cytoskeleton with other cell organelles within a live macrophage. The acoustic radiation force associated with ultrasound (US) pulses displaces an integrin-bound MB without detaching it, inducing a strain to the cell via the RGD-integrin-cytoskeleton linkage.

We employed acoustic tweezing cytometry (ATC)^13,14^ as a new approach for *in situ* mechanical characterization of macrophages. In ATC, lipid-encapsulated microbubbles (MBs) were functionalized to target to integrin receptors of macrophages. Biocompatible and highly responsive to ultrasound (US) excitation^15,16^, encapsulated gaseous MBs have been used as an imaging contrast agent for clinical US imaging^17^. In ATC, an US pulse is applied to generate a net force, i.e., the primary acoustic radiation force, on a MB in the direction of acoustic wave propagation as the result of momentum transfer^18^. This directional force is exploited for displacing integrin-anchored MBs without detachment^13,14^, thereby generating controlled deformation by extending the MB-integrin-cytoskeleton linkage in the cells (Fig. 1)^13,14^. The displacement of integrin-anchored MB thus represented deformation under a controlled force. During the US pulse, the force was constant, thereby enabling a creep test for assessment of the viscoelastic characteristics of the MB-integrin-cytoskeleton linkage from the displacement of integrin-anchored MB. Specifically in this study, we measured the time dependent displacement of the integrin-bound MB due to ATC application, and from which we calculated parameters including cell compliance, defined as MB displacement divided by the force acting on the MB, residual displacement after removal of the acoustic radiation force, as well as the creep and recovery time constants. These parameters describe the characteristic behavior observed in conventional creep testing and were used in this study to assess the viscoelastic characteristics of macrophages.

The acoustic radiation force acting on a MB by an US pulse depends a number of parameters including US frequency, acoustic pressure amplitude, and the size of the MB^18^. While US parameters may be chosen to avoid inducing irreversible changes to cells for characterizing cellular mechanical property, US pulses with higher acoustic pressures and longer duration will generate larger acoustic radiation force and large MB displacements, thereby inducing higher strain to the cells via the MB-integrin-cytoskeleton linkage and deforming the intracellular network within the cells. Since the CLDIs reside within the cytoskeleton network within macrophages, in this study, we explored the use of ATC to mechanically mobilize the rod-shaped CLDIs within macrophages via the integrin-bound MBs outside the cells for targeted destruction of macrophages to release the drug crystals.

## Materials and Methods

All methods in this study were performed in accordance with the University of Michigan guidelines and regulations.

### Clofazimine administration to mice

Mice (4 week old, male C57Bl6) purchased from the Jackson Laboratory (Bar Harbor, ME) were acclimatized for 1 week in a specific-pathogen-free animal facility. Clofazimine (CFZ) (C8895; Sigma, St. Louis, MO) was dissolved in sesame oil (Shirakiku, Japan) to achieve a concentration of 3 mg/ml, which was mixed with Powdered Lab Diet 5001 (PMI International, Inc., St. Louis, MO) to produce a 0.03% drug to powdered feed mix, and orally administered ad libitum for up to eight weeks. A corresponding amount of sesame oil was mixed with chow for vehicle treatment (control). For washout experiments, mice were fed the CFZ diet for eight weeks. Following eight weeks treatment, mice were placed on the vehicle diet for the following eight weeks. Mice were euthanized via carbon dioxide asphyxiation and exsanguination. Animal care was provided by the University of Michigan’s Unit for Laboratory Animal Medicine (ULAM), and the experimental protocol was approved by the Committee on Use and Care of Animals (Protocol PRO00005542).

### Peritoneal macrophage isolation

Following euthanasia, a small incision was made in the lower abdomen. The peritoneal cavity was then flushed 10 mL of ice cold DPBS containing 5% FBS (Sigma) and collected. The peritoneal lavage was centrifuged for 10 min at 400 × g, 4 °C, and then resuspended in DMEM media (Life Technologies) with 5% FBS and Penicillin/Streptomycin, counted, cells were plated onto 35 mm glass bottom dishes (Part No. P35G-2-14-C-GR50, MatTek Corporation, Ashland, MA) at a density of 50,000 cells. The cells were allowed to attach overnight and washed with media.

### CLDI isolation

Following eight weeks of drug treatment, mice were euthanized by carbon dioxide asphyxiation and exsanguination, and spleens were harvested. The tissue was diced and homogenized manually, and collected in PBS. The homogenate was then centrifuged (250 × g for 6 minutes) to remove large cellular debris. The supernatant was removed, and the pellet was washed two more times with PBS. CLDIs were further purified using a 3-layer discontinuous gradient (50%, 30% and 10% sucrose in DI water) centrifugation method (3220 × g for 60 min, no brakes). The CFZ content of the isolated CLDIs was determined using a plate reader at wavelength 450 nm following dissolution in 9M sulfuric acid followed by comparison with calibrated CFZ standards.

### Macrophage treatments

Following an overnight plating and washing, peritoneal macrophages were treated with either polyethylene beads (1–3 µm in diameter), or 5 µM soluble CFZ in DMSO, or 5 µM CLDIs suspended in DPBS in three experimental groups. After an overnight incubation, the media was removed, the cells were washed in DMEM, and fresh, non-drug containing media was placed into the dish prior to incubation with the microbubbles and treatment with ultrasound (US). For fixing the cells, macrophages were treated with z-fix (Anatech Ltd, Battle Creek, MI, USA) for 5 min before microbubble attachment and US application.

### Functionalization of microbubbles (MBs) and attachment of RGD-MBs to macrophages

Targesphere^TM^-SA lipid microbubbles (MBs) (diameter 2.61 ± 0.046 μm, mean ± SEM; 2.80 × 10^9^ mL^−1^; Targeson, San Diego, CA) were used in our study. To functionalize the MBs, were mixed with biotinylated RGD peptides (Cyclo[Arg-Gly-Asp-D-Phe-Lys(Biotin-PEG-PEG)] dissolved in diH_2_O at 2 mg mL^−1^; Peptides International, Louisville, KY) at a volume ratio of 5:1 for 30 min at room temperature for conjugation. The mixture was diluted 75X with culture medium before use. To attach the MBs with RGD (RGD-MBs) to the macrophages, culture medium was first removed, followed by two PBS washes. 50 μL of the diluted RGD-MBs solution was added to the petri-dish and incubated at 37 °C for 15 min with the petri-dish flipped upside down to allow RGD-MBs to float up and attach to the adherent cells via specific RGD-integrin binding. Unbounded RGD-MBs were removed with two gentle wash with culture medium before US application.

### US system for ATC

A 1.25 MHz planar transducer with a circular aperture (radius *α* = 3.18 mm, Advanced Devices, MA, USA) was used for US application. The US transducer was positioned at 45° angle to the horizontal direction facing downward towards the cells seeded on the petri-dish at a distance of 9 mm to ensure far field condition to avoid rapid varying axial acoustic field. This distance was determined by the distance of the last axial maximum from the transducer surface, *Z*_*lam*_ = *a*^2^/*λ*, where *a* is the radius of the transducer and *λ* is the wavelength. Similar to Rayleigh distance, *Z*_*lam*_ is the distance where the field transitions from near field to far field. For our transducer with radius of 3.18 mm and center frequency of 1.25 MHz, *Z*_*lam*_ = 8.4 mm. The active surface of the transducer was fully submerged in the culture medium in the petri-dish to allow acoustic coupling. The petri-dish was placed on a heating place at 37 °C. The US transducer was driven by a waveform generator (33220 A; Agilent, Santa Clara, CA) and a power amplifier (75A250; Amplifier Research, Souderton, PA) and was calibrated using a hydrophone (HNR-0500; Onda, Sunnyvale, CA). The transducer has a −6 dB beam width of 3.54 mm at *Z*_*lam*_.

### Single pulse ATC for cell mechanical phenotyping

To characterize mechanical properties of macrophages, an US tone burst of 50 ms duration (62500 cycles) with an acoustic pressure of 0.025 MPa was applied to the macrophages attached with integrin targeted MBs. Real time video-microscopy was employed to record the MB activities before, during, and after application of the US pulse (a tone burst) using a high-speed camera (FASTCAM SA1, Photron, San Diego, CA) at a frame rate of 2000 frames/s. Images of the MBs were processed with an automated MATLAB script, and the MB diameter and location in each frame were extracted and tracked over time to obtain the MB displacement as a function of time. MB displacement reached maximum at the end of the US pulse (or tone burst) when t = 50 ms.

We then calculated J(t), which is the measured MB displacement divided by the primary acoustic radiation force. Therefore J(t) reached maximum also at the end of the US pulse when t = 50 ms, and we defined this maximum value of J(t) as the effective cell compliance. The primary acoustic radiation force was calculated using^18,19^:

1 $${F}_{P}=\frac{2\pi {P}_{A}^{2}D{R}_{0}}{{\delta }_{tot}{\rho }_{0}c{\omega }_{0}T}$$

where P_A_ is the acoustic pressure amplitude, D is the pulse duration (50 ms), R_0_ the bubble radius at equilibrium, δ_tot_ the total damping constant (δ_tot_ = 0.16), ρ_0_ the medium density (1000 kg m^−3^), and c the speed of sound in media (1500 m s^−1^), ω_0_ = 2πf_0_ (f_0_ is the frequency of the incident US pulse 1.25 MHz), and T is the pulse repetition period (1 s)^18^. Effective cell compliance (Max J(t)), was extracted from the time-dependent, normalized displacement curves for each cell. Percent recovery of the normalized MB displacement was calculated as:

2 $$ \% \,Recovery=\frac{Max(J(t))-J{(t)}_{residual}}{Max(J(t))}\ast 100 \% ,$$

where the nominator denotes the recovered normalized displacement. Creep and recovery time constants were calculated as the time needed for reaching 63% of the maximum J(t), and 63% of the recovered normalized displacement, respectively. A total of 24–35 cells were analyzed per treated group and 114 cells were analyzed for the untreated control group.

### ATC for cell destruction

To generate large MB movements and actuate the phagocytosed CLDIs, multiple US pulses with longer duration and acoustic pressure amplitude (10 US tone bursts or pulses, pulse repetition frequency 1 Hz, duration of each tone burst 500 ms, acoustic pressure 0.07 MPa) were applied to the cells (total duration of 10 s). To determine cell death, propidium iodide (PI) (100 µM, Sigma-Aldrich, St. Louis, MO, USA) was added in the DMEM before US application. Prior to the application of US, a bright-field image and fluorescence image (626 nm emission) for PI signal were captured. A second PI image was taken immediately following US application. Based on the bright-field image taken before US application, a region of interest comprising an entire cell was outlined, and this outline was used to measure the intracellular PI fluorescence intensity before and after the application of US. The ratio of the pre- and post- US PI fluorescence was determined. Live cells would have a ratio near 1, while the cells that were destructed by ATC will have ratio values greater than 1. Thirty cells per treatment group were analyzed.

Fast frame video-microscopy was employed to record bright field images of cells in real time using a high-speed camera (FASTCAM SA1, Photron, IN) at a frame rate of 2000 frames/s to monitor the dynamic movements of MBs and CLDIs within the macrophages. An automated MATLAB script was used to track the MB and phagocytosed CLDI movements. MB movements from 19 to 25 cells were analyzed per treatment group. Movements of 60 to 120 individual phagocytosed CLDIs were analyzed in terms of the rotation angle of the rod-like structure and center displacement.

### Statistical analysis

With the assumption that the distribution of the measured data is Gaussian, results were presented as mean ± standard error of mean, which was calculated as $\frac{\sigma }{\sqrt{n}}$, where *σ* is the standard deviation of the data and *n* the sample size for each data presented. Data were analyzed with one-way analysis of variance (ANOVA) followed by pair-wise t-test or Tukey’s honest significant difference (HSD) test. The difference is considered significant if *P* < 0.05.

## Results

### Time dependent integrin-bound MB displacements reveal viscoelastic characteristics of macrophages

For mechanical phenotyping of macrophages, experiments were performed using ATC with a single US tone burst (referred as an US pulse) of duration 50 ms (acoustic pressure 0.025 MPa) to displace integrin-bound MBs (Fig. 1). To ascertain the effects of CLDIs on macrophage mechanical properties, we conducted experiments to characterize mechanical properties of macrophages under several conditions as described in the materials and methods section.

**Figure 2:**
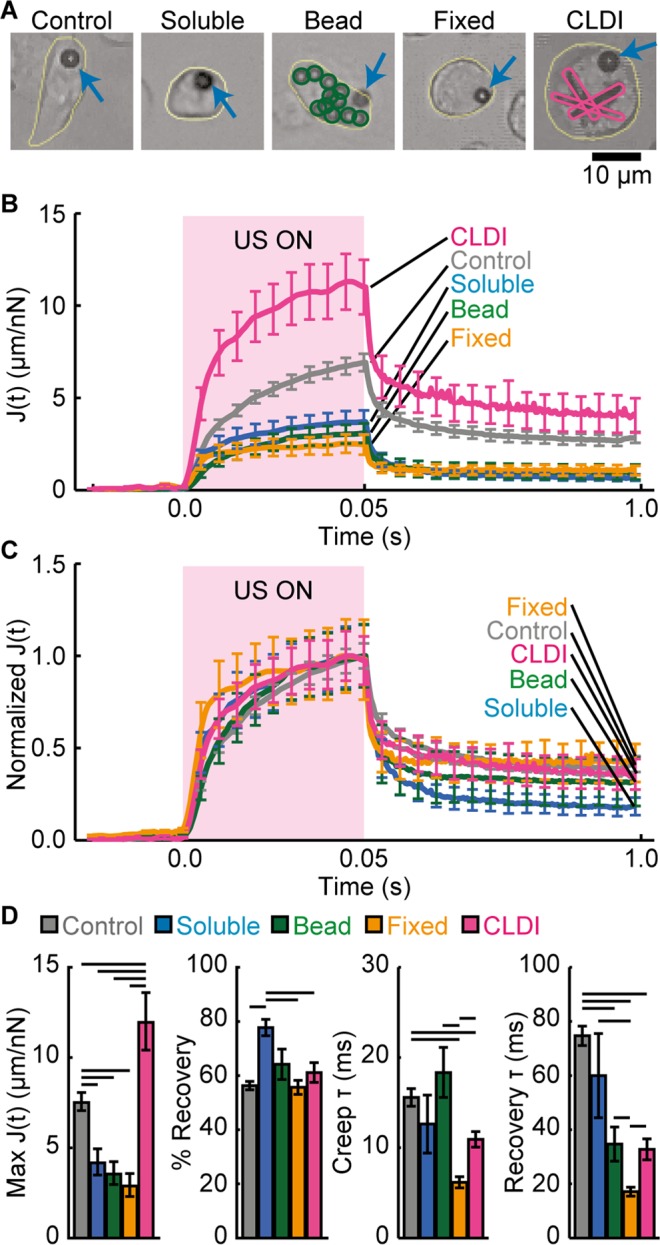
Characterization of mechanical property of macrophages using ATC. (**A**) Five groups of macrophages: control, treated with soluble CFZ, loaded with polyethylene beads, fixed, and loaded with CLDIs. Cells are outlined in yellow. Blue arrows point to MBs. Polyethylene beads are outlined in green, CLDIs in pink. (**B**) J(t). (**C**) Normalized J(t). (**D**) Effective compliance (Mean ± S.E., n = 24–35 cells per treatment group, n = 119 for control group), percentage of recovery, creep and recovery time constants (extracted from averaged J(t), n ≥ 4). Time constants are defined as the time needed for reaching 63% of maximum value of J(t) for creep, and 63% of recovered J(t) for recovery portion respectively. (Line above the bars indicates *p* < 0.05 in paired *t* test).

Our results from these experiments show that integrin-anchored MBs attached to macrophages in all 5 groups (control, macrophages treated with soluble CFZ, macrophages with phagocytosed polyethylene beads, fixed macrophages, and macrophages with CLDIs) responded effectively to US application (Fig. 2A). Clearly, during the US pulse (t = 0–50 ms), the integrin-bound MBs were displaced away from their pre-US locations (Movies S1–S5), thereby exerting strains to the cell by extending the MB-integrin-cytoskeleton linkage. As the acoustic radiation force acting on a MB was constant during the US pulse, ATC enables a creep test of cell mechanical properties *in situ*. After the end of US pulse, the displaced MBs retracted back towards their pre-US locations, indicating strain relaxation in the distorted linkage (Fig. 2B).

For analysis, we first determined J(t), which was calculated as the measured displacement of an integrin-bound MB divided by the acoustic radiation force acting on the MB. The effective cell compliance was calculated as the J(t) value at the end of the US pulse when the MB displacement reached maximum. Normalized J(t) was obtained by dividing J(t) by its maximum value (reached at the end of US pulse) (Fig. 2B,C). It is clear that the time-dependent behavior of J(t) for macrophages in all groups exhibited characteristic viscoelastic behavior, sustaining an instantaneous elastic increase immediately after US application, followed by a time-dependent creep process during the US pulse. Recovery after the end of US pulse exhibited a rapid elastic recovery, followed by a slower process that often left a small, permanent deformation (Fig. 2B,C). These viscoelastic characteristics of integrin-bound MB displacements represented the mechanical property of the macrophages.

To accommodate microscopic imaging with an inverted microscope and more importantly to avoid artifacts of US reflection and interferences directly from the bottom of the cell culture dish in our experiments, the US transducer was oriented at an angle (i.e. 45°) with the horizontal plane so that the acoustic radiation force was also pointing in this direction with a 45° angle. Since we measured movement of integrin-bound MBs within the horizontal plane microscopically in this study, the force responsible for the measured MB displacements was the horizontal projection of the acoustic radiation force described in Eq. (1).

### CFZ CLDIs softens macrophages and increases their elasticity

Plots of J(t) show that cell fixation, phagocytosis of polyethylene beads, and soluble CFZ treatment rendered macrophages stiffer as shown by the smaller MB displacements under the influence of US pulse with the same condition (Fig. 2B). However, CFZ CLDIs had the opposite effects on macrophages: the macrophages loaded with CFZ crystals were much softer, with the largest MB displacements sustained under the same US condition compared to the other groups (Fig. 2B).

The maximum value of J(t) from each group, represented an effective compliance of the cells, or the inverse of modulus that may be used to gauge macrophage stiffness (Fig. 2D). Compared to control cells (compliance 7.12 ± 0.47 µm/nN; *n* = 114), macrophages treated with soluble CFZ exhibited a significant decrease in compliance (3.77 ± 0.64 µm/nN; *n* = 34), and phagocytosis of solid polyethylene had similar effects on macrophages with a much decreased compliance (3.18 ± 0.54 µm/nN; *n* = 35), indicating that soluble CFZ treatment and phagocytosis of polyethylene beads made the macrophages stiffer (Fig. 2D). As expected, fixed cells were stiffest with the smallest compliance (2.50 ± 0.48 µm/nN; *n* = 25), due to the use of cross-linking agent (Z-fix) in fixation treatment. On the contrary, macrophages loaded with CLDIs became notably softer, with a much higher compliance (11.42 ± 1.55 µm/nN; *n* = 24) than control cells (Fig. 2D). There were no correlations between compliance of the CLDI− loaded cells vs. cell size or CLDI quantity within the cells (Fig. S1), thus the changes in compliance were unlikely related to these factors but likely to the presence of CLDIs within the macrophages. The observed opposite effects on macrophage stiffness likely reflect the different mechanisms involved in the different processes or treatments to macrophages. Our data indicate that macrophage stiffness is sensitive to different treatments the macrophages were exposed to and thus the macrophage state. Our observation that macrophages with CLDIs became softer is consistent with literature reports that Young’s modulus measured using AFM decreased in stimulated macrophages compared to resting macrophages^20^. Softening of macrophages following stimulation may be attributed to the changes of cytoskeleton structure.

Other parameters were also obtained from the measured J(t) to assess viscoelastic characteristics of macrophages. Fixed macrophages exhibited a pronounced instantaneous increase in J(t) immediately after application of US pulse, and behaved more like an elastic solid compared to control cells. The increased elasticity for fixed macrophages was also evident from a much faster creep (time constant 6.66 ± 0.75 s; *n* = 5) and recovery (time constant 19.66 ± 2.93 s; *n* = 5) for these macrophages than control cells (creep time constant 15.43 ± 0.96 s; *n* = 22 and recovery time constant 73.47 ± 3.69 s; *n* = 22) (Fig. 2D). Treatment with soluble CFZ and loading of polyethylene microbeads did not change the creep time constant significantly, suggesting no significant change in elasticity. However, the presence of CLDIs inside macrophages significantly decreased creep time constant (10.62 ± 0.81 s; *n* = 4) and recovery time constant (32.73 ± 3.37 s; *n* = 4) compared to control cells (creep time constant 15.43 ± 0.96 s; *n* = 22, and recovery time constant 73.47 ± 3.69 s; *n* = 4), suggesting increased elasticity due to phagocytosis of CFZ biocrystals (Fig. 2D). Since macrophage elasticity is linked to its activation and function^3^, CFZ biocrystals may impact activation of these macrophages with CLDIs^5^.

### Cytotripsy by ATC actuation of CLDIs in macrophages as intracellular microblender

**Figure 3:**
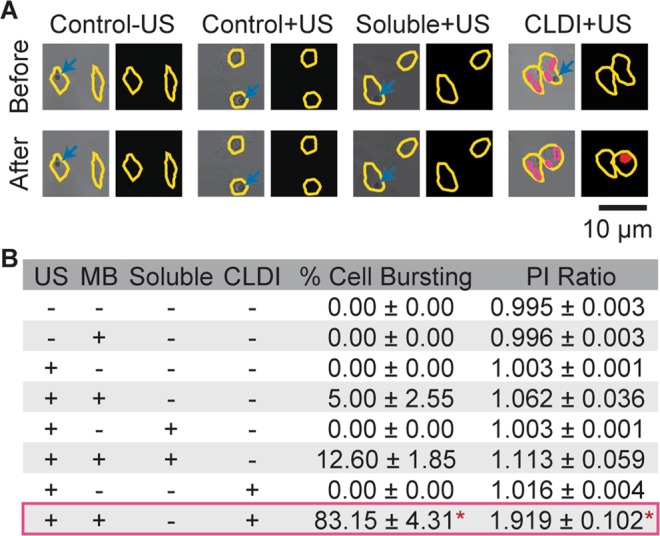
ATC-induced destruction of macrophages with CLDIs. (**A**) Macrophages in 4 different groups before- (left, bright field image) and post-ATC (right, fluorescent image). Cell border is outlined in yellow, blue arrows denote MBs and CLDIs were outlined in pink. US application induced macrophages containing CLDIs to burst, indicated by propidium iodide (PI) fluorescence. (**B**) Percentage of cell destruction (determined from videomacroscopy, n = 3 per group) and ratio between post-US and pre-US PI fluorescence intensity (n = 30–35 per group). *p < 0.05, ANOVA, Tukey’s HSD.

The effective MB-integrin-cytoskeleton linkage results in application of strains to the cells via displacement of MBs during ATC application, distorting intracellular structures within the macrophages. In particular, here we employed ATC to generate irreversible changes to macrophages by actuating CLDIs within macrophages using multiple US pulses (1 Hz pulse repetition frequency) at higher acoustic pressure (0.07 MPa) and longer duration (500 ms). Notably, application of ATC with these US pulses resulted in effective and selective destruction of CLDI− loaded macrophages, without affecting other macrophages without CLDIs (Fig. 3). Cytotripsy of macrophages resulted in release of the CLDIs from the host macrophages.

**Figure 4:**
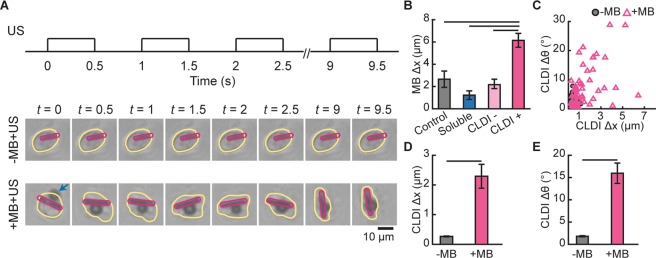
MB and CLDI movement induced by ATC for cytotripsy. (**A**) Applied US pulses for cytotripsy. Pulse duration 500 ms, PRF 1 Hz, and acoustic pressure 0.07 MPa. Images show CLDI− containing peritoneal macrophage exposed to US with (+) and without (−) MB attached to integrin of the cells. Cell border is outlined in yellow, CLDI in pink, and blue arrow points to the MB. (**B**) Maximum MB displacements achieved during the first US pulse for control, soluble CFZ-treated, CLDI− and CLDI+ macrophages. Macrophages cultured with CLDIs without and with CLDI phagocytosis are denoted as CLDI− and CLDI+, respectively. (Mean ± S.E., n = 19–25 cells per group, lines showing *p* < 0.05, ANOVA). (**C**) Total angle of rotation vs. displacement of the center of mass of individual CLDI in macrophages with and without attached MBs. (**D,E**) Bar plot of individual CLDI displacements (**D**) and rotation angle (**E**) during the first US pulse for CLDI− containing macrophages with and without MBs. (Mean ± S.E., n = 69 and 111 for −MB + US and +MB + US groups respectively, lines showing *p* < 0.05, paired t test).

To investigate how ATC induced targeted cytotripsy, we examined MB displacements and CLDI movements, as well as morphological changes in macrophages during ATC application. Fast frame video-microscopy showed that application of ATC induced significant movements of MBs and mobilized CFZ biocrystals within macrophages (Fig. 4A, Movie S6). The large and rapid movements of rod-shaped CFZ crystals within the macrophages turned the CFZ crystals into an intracellular microblender which generated significant changes in cell shape (Fig. 4A), followed by cell destruction within seconds of US application.

Quantification of the movements of MBs and CLDIs revealed that during the first US pulse (duration 500 ms) (Fig. 4A), total displacement of a MB attached to a macrophage with CLDIs (6.15 ± 0.62 µm; *n* = 21) were 200–300% of the displacements of MBs attached to other macrophages without CLDIs (control cells: 2.66 ± 0.75 µm, *n* = 29; cells treated with soluble CFZ: 1.22 ± 0.39 µm, *n* = 25; cells without CLDIs after incubation with the crystals (CLDI−): 2.17 ± 0.44 µm, *n* = 19) (Fig. 4B). The observation of larger MB displacements in macrophages with CLDIs is consistent with our finding that macrophages loaded with CLDIs were much softer (Fig. 2D), which allowed larger MB displacements to occur when macrophages are less stiff.

The large displacements of integrin-bound MBs resulted in significant CLDI movements within the host macrophages. The center displacement of the rod-shaped CFZ crystals within macrophages was 2.29 ± 0.41 µm (*n* = 69) and their rotation angle 15.96 ± 2.30 degree (*n* = 69), in contrast with the minimal center displacement of the crystals inside the cells without MBs (0.26 ± 0.01 µm; 1.8 ± 0.14 µm; *n* = 111) (Fig. 4C–E), suggesting mobilization of the CLDIs within macrophages by enhanced actuation of the integrin-bound MBs by US pulses at higher acoustic pressure and longer duration.

Taken together, these data demonstrate a phenomenon of mechanopharmaceutical cytotripsy by mobilizing CLDIs as intracellular micro-blender within macrophages via acoustic actuation of integrin-bound MBs, resulting in release of CFZ biocrystals from these CLDI− loaded macrophage without affecting other cells.

## Discussion

Results obtained from this study demonstrate the utility of ATC as a new and versatile technique for both *in situ* mechanical phenotyping of macrophages and targeted cytotripsy.

Conventional techniques such as micropipette aspiration^21^, optical tweezer^22^, and atomic force microscopy (AFM)^23,24^ apply localized forces to single cells to characterize cellular mechanical properties. However, these techniques are generally not amenable for assessing an ensemble of cells simultaneously. AFM may require expensive equipment and special sample preparation. Magnetic twisting cytometry (MTC) generates twisting stress to multiple cells using functionalized magnetic microbeads attached to cells^25–27^. But solid microbeads are difficult to remove from the cells, which may limit downstream analysis or continued use of the treated cells. In contrast, ATC has a number of advantages for probing cell mechanical property *in situ*^28^. It uses lipid-encapsulated MBs that are biocompatible and may be easily removed from cells without leaving behind exogenous materials. Suitable for *in situ* assessment of cellular mechanical property^28^, ATC does not require extensive sample preparation or expensive system.

Our results show that macrophage mechanical property is sensitive to the treatment they were subjected to, an observation that is consistent with previous findings that macrophage elasticity and phagocytosis are influenced by their mechanobiochemical environment^5^. Specifically, our results show that phagocytosis of CFZ biocrystals softened macrophages and increased their elasticity, opposite from soluble CFZ treatment and phagocytosis of solid polyethylene beads, suggesting that macrophage mechanical property reflect not only their mechanochemical environment but also the tensegrity of the cytoplasmic structures/processes inside the cells that are influenced by treatment such as phagocytosis of CFZ biocrystals.

The integrin-anchored MBs via RGD-integrin binding were connected to the intracellular molecules and structures such as focal adhesion (FA) and cytoskeleton. Thus the integrin-bound MBs may transmit the externally applied forces by US pulses into the cytoplasmic domain, capable of eliciting responses from mechanosensitive cells. For example, ATC that used multiple tone bursts has been shown to increase cellular contractile forces^13^, enhance osteogenic differentiation of human mesenchymal stem cells (hMSCs)^29^, improve survival of human embryonic stem cells (hESCs)^30^ or initiate differentiation of hESCs^31^. To minimize these cellular effects, in this study, we used single tone burst in ATC to assess cellular mechanical property.

Previously, AFM has been utilized to assess mechanical property of cells^24,32^ including macrophages^20,33,34^. Used as an indentation method via the compression of cantilever onto the cell membrane and measurement of ensuing deformation of the cell surface, Young’s modulus of macrophages were obtained to be in a wide range of values^20,33,34^, from 0.8 kPa up to 60 kPa, depending on factors such as surface coating and incubation time. Higher values of measured Young’s modulus has been attributed to the size of the cantilever tip used in AFM, when a smaller tip may lead to locally occurring strain hardening^24^.

Compliance of macrophage measured using ATC in this study was based on the acoustic radiation force induced stretch of MB-integrin-cytoskeleton linkage, which was analogous to a mass spring system. Macrophage compliance measured using ATC is the equivalent of the inverse of the spring constant. While cell membrane and other structures within macrophage could also affect the MB displacement, we expect that compliance measured using ATC primarily depends on the MB-integrin-cytoskeleton linkage. Young’s modulus measured using AFM included the collective contribution of cell membrane, cell cortex, cytoskeleton, and even organelle^24^. Thus direct comparison of our measured cell compliance with reported values of Young’s modulus using AFM is not available unless some assumptions are made. Applying beam theory, and assuming values of the length of the MB-integrin-cytoskeleton linkage affected by ATC (e.g. 10 µm) and radius of the bean (e.g. 0.05 μm), a compliance of 7.5 nN/μm for macrophages (Fig. 2D) may be converted to a Young’s modulus of 42.5 kPa, a value within the range of reported Young’s modulus of macrophages^20,33,34^. It is important to keep in mind that such conversion depends on the assumed values of length and cross section of the integrin-cytoskeleton linkage structure affected by ATC application.

Force transmission from MBs during ATC to intracellular domain of macrophages was seen via the phenomenon of CLDI mobilization within macrophages. Movements of the integrin-bound MBs outside the cells by ATC application resulted in forces and torques on the rod-shaped crystals nested within the intracellular network, effectively turning them into intracellular microblender to disrupt the intracellular structures and disintegrate the cells. This new phenomenon of acoustic mechanopharmaceutical cytotripsy releases the CLDIs from macrophages, and may provide a new strategy for macrophage-directed drug delivery^35,36^.

## Supplementary information

Supplementary Information

Movie S1

Movie S2

Movie S3

Movie S4

Movie S5

Movie S6

Movie S7

Movie S8

Movie S9

Movie S10

Movie S11
