# Supplementary material for: Acoustic tweezing cytometry for mechanical phenotyping of macrophages and mechanopharmaceutical cytotripsy
Source: Sci Rep. 2019 Apr 5;9:5702. doi: 10.1038/s41598-019-42180-3 (PMC6450871; doi:10.1038/s41598-019-42180-3)
Supplement: Supplementary file 1 — Supplementary Information [file 41598_2019_42180_MOESM1_ESM.pdf]

# Acoustic tweezing cytometry for mechanical phenotyping of macrophages and mechanopharmaceutical cytotripsy

Xiaowei Hong,[1,#] Phillip M. Rzeczycki,[2,#] Rahul K. Keswani,[2] Mikhail D. Murashov,[2]
Zhenzhen Fan,[1] Cheri X. Deng[1,3,*] and Gus R. Rosania[2,*]

Supplemental movies

Movie S1 – S5:

ATC for probing the mechanical properties of control macrophages (S1), macrophages treated with soluble CFZ (S2), macrophages loaded with polyethylene microbeads (S3), fixed macrophages (S4) and macrophages loaded with CLDIs (S5)

Movie S6 – S11:

ATC for cytotripsy: macrophages loaded with CLDIs with microbubble (MB) attached to the cell exposed to cytotripsy pulses(S6); control macrophage without MB (S7); control macrophage with MB (S8), macrophage treated with soluble CFZ without MB (S9); macrophages treated with soluble CFZ with MB (S10); macrophages loaded with CLDI without MB (S11).

Supplemental Figures:

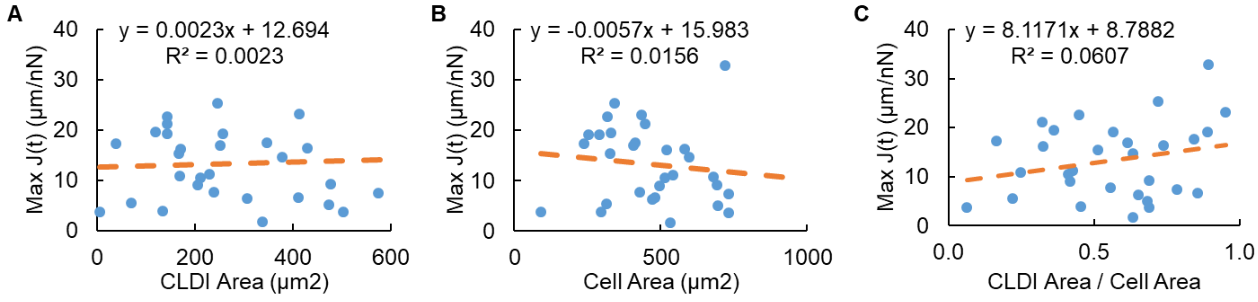


Supplemental Figure S1. Correlation between area of CLDIs within a macrophage vs. macrophage compliance (maxJ(t)), cell area vs. macrophage compliance, as well as (CLDI area)/(cell area) vs. macrophage compliance.  No strong linear correlation was found between these factors.
